# Notes and Notices

**Published:** 1888-01

**Authors:** 


### NOTES AND NOTICES.

REMOVED.—D. C. McLaren, M. D., has removed from Brantford, Canada, to Nashville, Michigan. Dr. McLaren is one of our most energetic and earnest homœopaths, and hence we give him a hearty welcome to the United States.

THE CALIFORNIA HOMŒOPATH, with its January issue begins its sixth volume, and appears henceforth as a monthly. Professor W. A. Dewey will assist in conducting the journal. Success to it.

